# Improved long‐term outcomes after innovative preoperative evaluation and conception of precise surgery for gallbladder cancer

**DOI:** 10.1002/cam4.6513

**Published:** 2023-09-14

**Authors:** Zi‐Yao Jia, Yi‐Di Zhu, Xiang‐Song Wu, Jing‐Xiao Yang, Wen‐Guang Wu, Xu‐An Wang, Min He, Hui Wang, Lin‐Hua Yang, Jie Zhang, Xue‐Chuan Li, Lu Zou, Huai‐Feng Li, Fei Zhang, Run‐Fa Bao, Xu‐Ya Cui, Xiao‐Ling Song, Wei Chen, Wei Gong, Mao‐Lan Li, Ying‐Bin Liu

**Affiliations:** ^1^ Department of Biliary‐Pancreatic Surgery, Renji Hospital, School of Medicine Shanghai Jiao Tong University Shanghai China; ^2^ Shanghai Key Laboratory of Biliary Tract Disease Research Shanghai China; ^3^ Shanghai Research Center of Biliary Tract Disease Shanghai China; ^4^ Department of General Surgery Xinhua Hospital Affiliated to Shanghai Jiao Tong University School of Medicine Shanghai China; ^5^ State Key Laboratory of Oncogenes and Related Genes Shanghai China; ^6^ Shanghai Cancer Institute Shanghai China

**Keywords:** 3D visualization preoperative evaluation, enhanced recovery after surgery, gallbladder cancer

## Abstract

**Background:**

Three‐dimensional visualization preoperative evaluation **(**3D‐VPE) and enhanced recovery after surgery (ERAS) have been suggested to improve outcomes of cancer surgery in patients, yet little is known regarding their clinical benefit in patients with gallbladder cancer (GBC). We hypothesized that the combination of 3D‐VPE and ERAS would improve the outcome of patients undergoing surgery for GBC.

**Objective:**

This study aimed to determine if 3D‐VPE and ERAS can improve the outcomes and overall survival in patients with GBC, establishing a novel patient management strategy for GBC.

**Methods:**

A total of 227 patients with GBC were recruited and divided into two groups: those who received traditional treatment between January 2000 and December 2010 (*n* = 86; the control group) and those who underwent 3D‐VPE and ERAS between January 2011 and December 2017 (*n* = 141). Univariate and multivariate analyses were employed to assess the relationship among disease stages, lymph node invasion, and cell differentiation between the two groups. Cox regression analysis was used to investigate patient survival in these groups.

**Results:**

Patients who underwent 3D‐VPE and ERAS showed a significantly higher R0 resection rate (67.4% vs. 20.9%, *p* < 0.001) and dissected lymph node number (26.6 ± 12.6 vs. 16.3 ± 7.6 *p* < 0.001) compared to the control group. The median survival was 27.4 months, and the 1‐ and 3‐year survival rates were 84.4% and 29.8%, respectively, in patients who received combined management; in the control cohort, the median survival was 12.7 months, and the 1‐ and 3‐year survival rates were 53.5% and 15.1%, respectively. In addition, some postoperative complications and risk factors were diminished relative to the traditionally treated patients.

**Conclusion:**

The implementation of 3D‐VPE and ERAS can significantly improve the prognosis and outcomes of patients with GBC and should be considered for wide use in clinical practice.

## Abstract 2

This is a retrospective study of patients with gallbladder cancer (GBC) treated at our institution over the course of 17 years. We show that three‐dimensional visualization preoperative evaluation and enhanced recovery after surgery can significantly improve prognosis and outcomes of patients with GBC and should be considered for wide use in clinical practice.

## 1 INTRODUCTION

Gallbladder cancer (GBC), an aggressive adenocarcinoma, accounts for 80%–95% of biliary tract cancers and constitutes 1.2% of all cancer deaths.^1^, ^2^ In China, 339 national centers of tumor registry have reported that approximately 11,200 new cases with an incidence rate of 3.90 out of 100,000 are (annually?) diagnosed with gallbladder and extrahepatic bile duct cancer. A study with 3528‐patient cohort from our center showed that the 5‐year overall survival rate (5yOS) of GBC patients was only 23.0%. Despite the advances in hepatobiliary imaging techniques, GBC is commonly diagnosed at an advanced stage^3^, ^4^, ^5^ with notable jaundice.^6^, ^7^ Surgical resection is currently the only effective treatment for patients with GBC, as the efficacy of adjuvant treatment is minimal.^8^, ^9^, ^10^ Given the anatomical structure of the gallbladder, which is intimately associated with the liver and other digestive organs and tissues, radical resection remains challenging. With the rapid development of medical technology and the gradual rise in the performance of precision surgery in the past few decades, patient prognosis in many diseases has significantly improved. The application of the recently developed three‐dimensional (3D) visualization technology for preoperative assessment has received considerable global attention in clinical practice.^11^ 3D visualization preoperative evaluation (3D‐VPE) involves evaluating the anatomical signature of blood vessels, the distribution of bile ducts adjacent to the liver, and the possibility of removing part of the liver, offering a precise approach to surgery. Enhanced recovery after surgery (ERAS), which involves preoperative counseling, perioperative nutrition, oral intake, and fluid management,^12^, ^13^ can decrease the incidence of postoperative complications in patients.^14^, ^15^, ^16^, ^17^ Here, we hypothesized that the combination of 3D‐VPE and ERAS improves the disease outcome of patients that undergo surgery. A retrospective study with patients who received 3D‐VPE and ERAS showed significantly higher R0 resection rate and dissected lymph node number than those in the control group. The median survival time and the 1‐ and 3‐year survival rates were also significantly improved in 3D‐VPE and ERAS‐treated patients. We propose that this combined management be used routinely in clinical practice with the expectation that it would improve patient care and quality of life.

## 2 METHODS

### 2.1 Patients

We enrolled a total of 227 eligible patients with GBC that underwent treatment between January 2000 and December 2017 in the department of surgery at Xinhua Hospital, Shanghai Jiao Tong University School of Medicine. All patients provided informed consent for the participation in this study.

### 2.2 Inclusion and exclusion criteria

The inclusion criteria included (1) pathologically proven GBC; (2) patients that were treated with cholecystectomy, radical cholectectomy, or GBC expanded radical operation; (3) patients that experienced enhanced‐computed tomography (CT), magnetic resonance imaging (MRI), magnetic resonance cholangiopancreatography (MRCP), and/or 3D visualization preoperative evaluation; or (4) patients that received ERAS management. The exclusion criteria were (1) lack of inageological examination; (2) lack of baseline characteristics including age, sex, gallstones, jaundices, albumin, AFP, CA19‐9, TNM stage, pathological diagnosis; (3)lack of perioperative and intraoperative management information; or (4) loss of follow‐up medical history.

### 2.3 Preoperative management

Before 2011, two‐dimensional (2D) evaluation was performed routinely. All patients underwent enhanced computed tomography (CT), magnetic resonance imaging (MRI), and/or magnetic resonance cholangiopancreatography (MRCP). To clarify the relationship between the tumor and adjacent major blood vessels, CT angiography (CTA) is performed. The surgery was mainly dependent on the findings on 2D evaluation and on individual surgeon experience.

**FIGURE 1:**
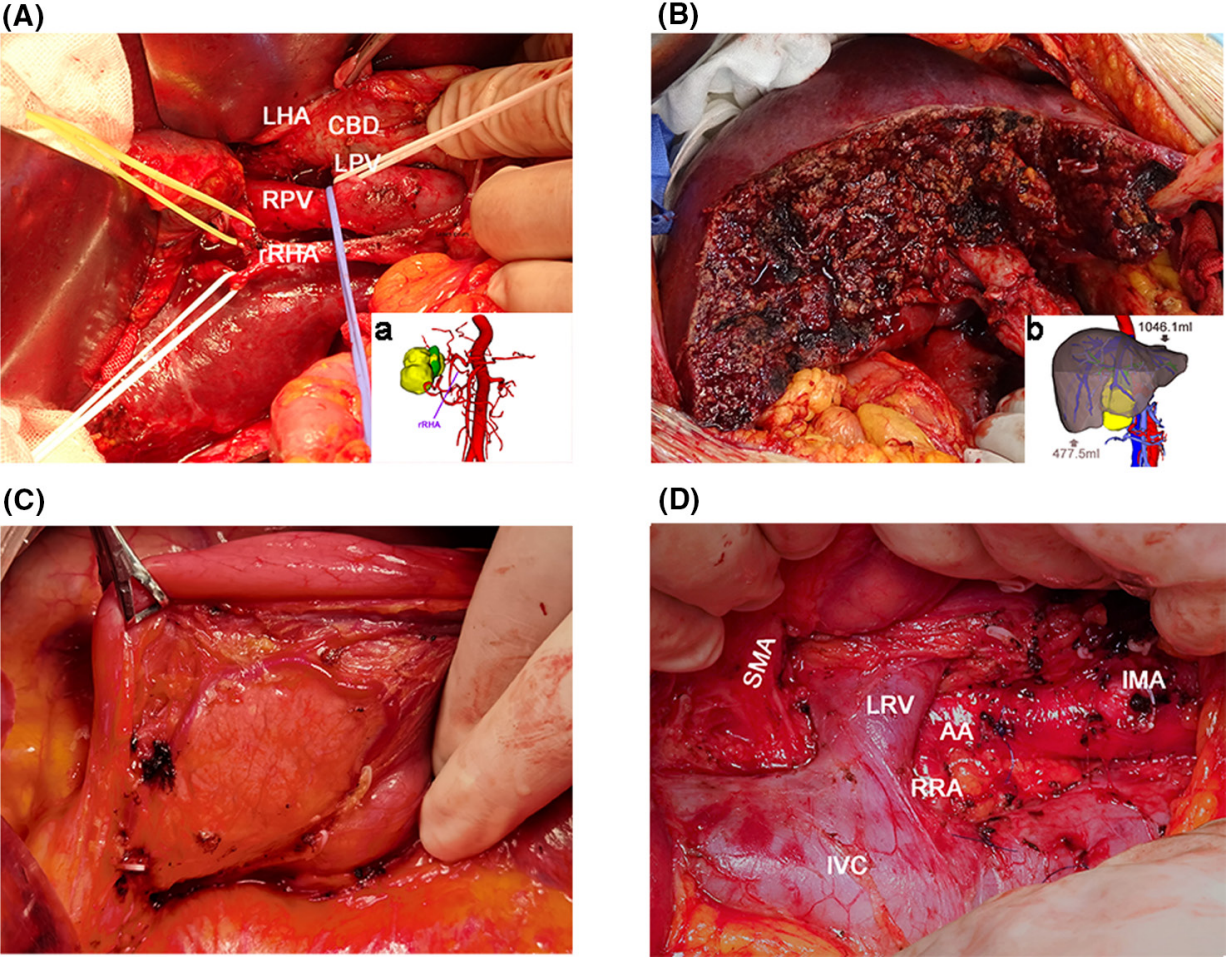
Application of 3D‐VPE in gallbladder cancer surgery. (A) Variant right hepatic vein found on 3D‐VPE; (B) the scope of segmental resection is based on 3D‐VPE; (C) dissection of lymph node No. 13a; (D) dissection of lymph node No. 16; (A‐a) 3D reconstruction of tumor infiltration and adjacent artery; (B‐b) residual liver volume calculation based on 3D‐VPE (resected liver volume is 477.5 mL, and residual liver volume is 1046.1 mL). AA, abdominal aorta; CBD, common bile duct; IMA, inferior mesenteric artery; IVC, inferior vena cava; LHA, left hepatic artery; LPV, left branch of portal vein; LRV, left renal vein; rRHA, replaced right hepatic artery, RPV, right branch of portal vein; RRA, right renal artery; SMA, superior mesenteric artery.

**FIGURE 2:**
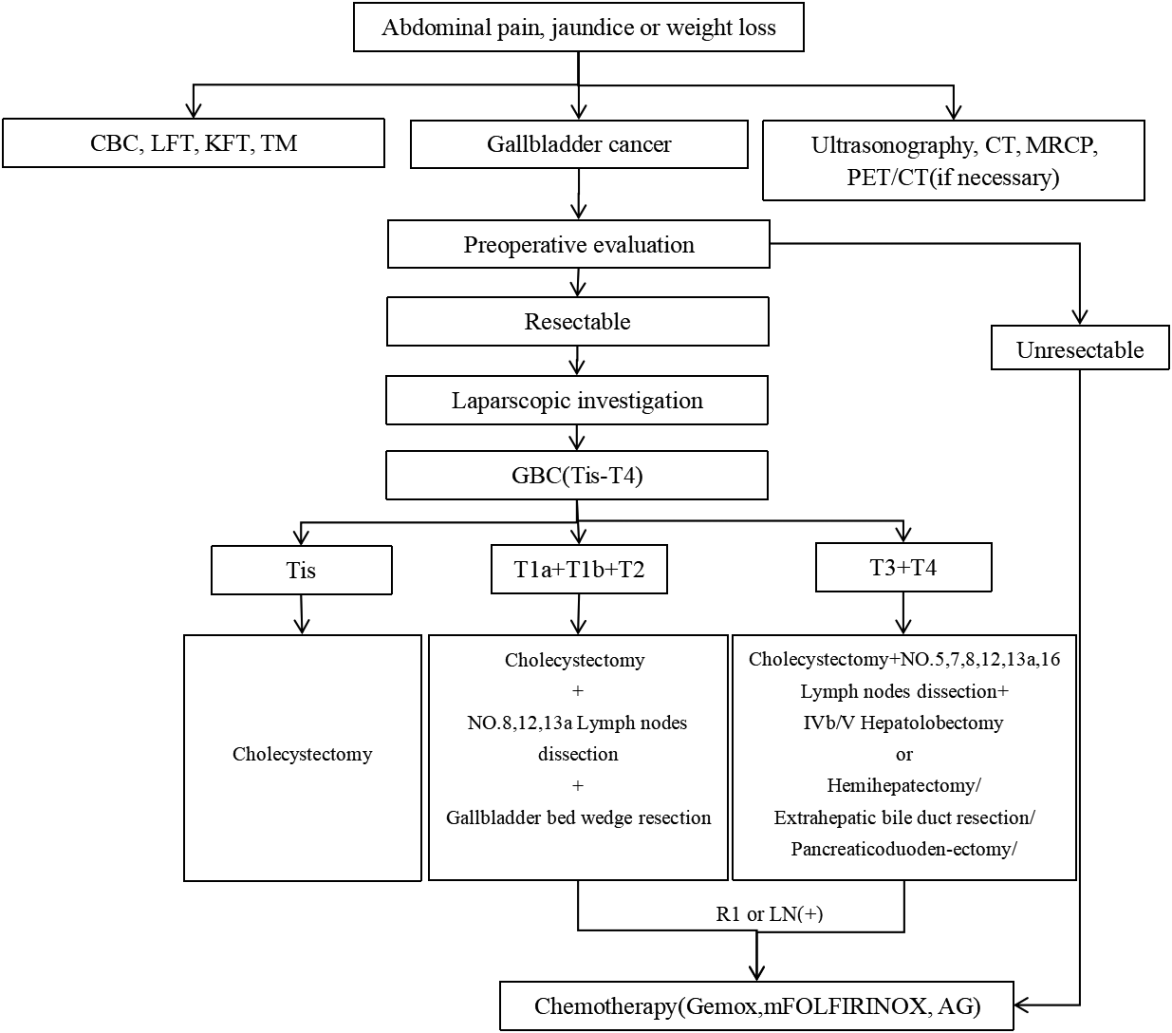
According to TNM staging based on the eighth edition of the AJCC staging manual, specific surgical procedures were performed.

After 2011, a 3D visualization system was used preoperatively and displayed intrahepatic and extrahepatic blood vessels and bile duct anatomy, the adjacent tissue associated with the tumor, the infiltration degree, and volume of the residual liver (Figure 1). An individualized surgical plan was established based on findings on 3D visualization and staging according to the TNM Classification of Malignant Tumours (TNM). The more reasonable and controllable surgical interventions were optimized by (1) determining the R0 resection margin and thus, confirming whether partial or full hepatectomy is needed to achieve successful R0 resection; (2) assessing anatomical relationships between major vessels and adjacent bile ducts to avoid damage to blood supply, ensure complete blood flow to the liver remnant, and prevent major intraoperative bleeding and postoperative bile leak; (3) determining the resection margin due to lymph node metastases, which is common in GBC and is an independent prognostic factor in patients^29^, ^30^, ^31^, ^32^ (regional lymph node dissection is critical for successful R0 resection); (4) systematically assessing surgical risks and control strategies; and (5) choosing surgical procedures and techniques depending on the stage of GBC (Figure 2).

### 2.4 Perioperative management

Before 2011, an abdominal drainage tube was inserted, which was removed at least 7 days after surgery, and commencement of enteral nutrition was late. After 2011, patients received ERAS management, which included

1. Patients were received elaborate preoperative education for better compliance;
2. Fasting solid foods for 6 h and fluid for 2 h before surgery, carbohydrate loading at 12 h before surgery;
3. Removal of some or all drainage tubes within 7 days after surgery and under allowable conditions,
4. Postoperative restricting the fluid intake,
5. Effective pain management,
6. Early enteral nutrition and oral feeding within 12 h after surgery.
7. Patients were encouraged to ambulate early (within 12 h) after surgery to reduce postoperative stress
8. Intravenous supplementation of albumin was administered to patients with low serum albumin levels (<30 g/L).

### 2.5 Follow‐up method

CT scanning and testing for tumor serological markers were routinely performed at outpatient clinics every 3 months to monitor the progress of the GBC and the liver condition. Long‐term follow‐up was carried out via telephone every month after surgery, and information on postoperative survival, disease recurrence, and continued treatment were obtained.

### 2.6 Statistics

A univariate analysis was conducted to compare patient characteristics, compliance variables, and outcomes between 3D‐VPE and ERAS protocol group and standard protocol group. Pearson chi‐squared or Fisher exact tests were used for categorical variables. The Kaplan–Meier method was used to estimate overall survival, which defined the time from first treatment or diagnosis to death from any cause. The log‐rank test was used to compare survival outcomes between the two groups. The restricted mean survival time (RMST) has been discussed extensively in survival analysis. Cox regression analyses were conducted to examine the associations among overall survival, demographics, and other covariates. All computations relied on the standard software (SPSS Statistics v24; IBM). A two‐sided *p* < 0.05 was considered statistically significant.

## 3 RESULTS

### 3.1 Baseline characteristics

The baseline characteristics of the patients in the absence (before 2011) and presence (after 2011) of 3D‐VPE and ERAS are shown in Table 1. A total of 227 patients with GBC between January 2000 and December 2017 were enrolled?, which involved 97 men and 130 women with an average age of 65 years (range: 44–87). In controls, 86 patients received traditional? treatment, while 141 patients experienced 3D‐VPE and ERAS management. For all patients, gallstones were frequently found in 140 (61.7%) patients and the common symptoms included right upper abdomen pain, nausea, vomiting, jaundice, anorexia, and weight loss. According to the eighth edition of the American Joint Committee on Cancer (AJCC) staging manual, 28 patients developed cancer with Stage I, 44 with Stage II, 109 cases with Stage III, and 46 cases with Stage IV. Histological analysis revealed that 68 cases were well differentiated, 89 cases moderately differentiated, and the rest 70 ones poorly differentiated. There were no significant differences in preoperative age, sex, presence or absence of gallstones, jaundice, albumin levels, alpha fetoprotein (AFP), and cancer antigen 19‐9 (CA19‐9) levels, TNM stage, and degree of differentiation between the two groups (*p* > 0.05).

**TABLE 1:** Baseline characteristics of patients in the two groups.

Characteristics	Total number of patients (% of total)	Number of patients in each protocol (%)		*p*‐value
		Standard	3D‐VPE and ERAS	
Age (y)				0.954
>60	142 (62.6)	54 (62.8)	88 (62.4)	
≤60	85 (37.4)	32 (37.2)	53 (37.6)	
Sex				0.334
Male	97 (42.7)	53 (38.4)	77 (45.4)	
Female	130 (57.3)	33 (61.6)	64 (54.6)	
Gallstones				0.770
Absent	87 (38.3)	34 (39.5)	53 (37.6)	
Present	140 (61.7)	52 (60.5)	88 (62.4)	
Jaundice				0.118
Absent	159 (70.0)	55 (64.0)	104 (73.8)	
Present	68 (30.0)	31 (36.0)	37 (26.2)	
Albumin				0.166
>35 g/L	19 (8.4)	10 (11.6)	9 (6.4)	
≤35 g/L	208 (91.6)	76 (88.4)	132 (93.6)	
AFP				0.678
>7 μg/L	18 (7.9)	6 (7.0)	12 (8.5)	
≤7 μg/L	209 (92.1)	80 (93.0)	129 (91.5)	
CA19‐9				0.447
>37 kU/L	130 (57.3)	52 (60.5)	78 (55.3)	
≤37 kU/L	97 (42.7)	34 (39.5)	63 (44.7)	
TNM stage				0.365
I	28 (12.3)	8 (9.3)	20 (14.2)	
II	44 (19.4)	19 (22.1)	25 (17.7)	
III	109 (48.0)	45 (52.3)	64 (45.4)	
IV	46 (20.3)	14 (16.3)	32 (22.7)	
pT				0.101
1	28 (12.3)	8 (9.3)	20 (14.2)	
2	58 (25.6)	28 (32.6)	30 (21.3)	
3	99 (43.6)	39 (45.3)	60 (42.6)	
4	42 (18.5)	11 (12.8)	31 (22.0)	
pN				0.389
0	97 (42.7)	38 (44.2)	59 (41.8)	
1	94 (41.4)	38 (44.2)	56 (39.7)	
2	36 (15.9)	10 (11.6)	26 (18.4)	
Differentiation				0.088
Well	68 (30.0)	33 (38.4)	35 (24.8)	
Moderately	89 (39.2)	31 (36.0)	58 (41.1)	
Poorly	70 (30.8)	22 (25.6)	48 (34.0)	

### 3.2 Intraoperative results

Patients who underwent surgery with standard protocol displayed a R0 resection rate of 67.4%, which was significantly higher than that in patients who underwent surgery with 3D‐VPE and ERAS protocol (20.9%, *p* < 0.001, Table 2). In addition, a greater number of lymph nodes were identified as positive and dissected in patients with 3D‐VPE and ERAS protocol (*p* < 0.001, Table 3). Of note, while the main complications included pulmonary and abdominal infections and hepatic dysfunction, there were no significant differences in the rate of their occurrence between the two periods (*p* > 0.05, Table 3). The incidence of biliary fistula and perioperative death was lower in patients who underwent 3D‐VPE and ERAS than in those who were treated with standard protocol (*p* < 0.05, Table 3).

**TABLE 2:** Resection and intraoperative pathways in the two protocols.

Characteristics	Total number of patients (% of total)	Number of patients in each protocol (%)		*p‐*value
		Standard	3D‐VPEand ERAS	
R0	113 (49.8)	18 (20.9)	95 (67.4)	<0.001
R1 or R2	114 (50.2)	68 (79.1)	46 (32.6)	
Conservative resection	41 (18.1)	11 (12.8)	30 (21.3)	<0.001
Radical resection	122 (53.7)	61 (70.9)	61 (43.3)	
Combined hemihepatectomy	32 (14.1)	8 (9.3)	24 (17.0)	
Combined pancreaticoduodenectomy	25 (11.0)	3 (3.5)	22 (15.6)	
Combined other organs resection	7 (3.1)	3 (3.5)	4 (2.8)	

**TABLE 3:** Comparison of surgical efficacy in the two protocols.

Characteristics	Protocol		*p*‐value
	Standard	3D‐VPE and ERAS	
Number of dissected lymph nodes	16.3 ± 7.6	26.6 ± 12.6	<0.001
Number of positive lymph nodes	4.4 ± 5.2	10.3 ± 9.7	<0.001
Postoperative complications (%)			
Biliary fistula	21 (24.4)	13 (9.2)	0.002
Pulmonary infection	23 (26.7)	21 (14.9)	0.002
Abdominal infection	10 (11.6)	11 (7.8)	0.075
Hepatic dysfunction	23 (26.7)	55 (39.0)	0.096
Perioperative death	5 (5.8)	3 (2.1)	0.036
One‐year survival rate (%)	46 (53.5)	119 (84.4)	<0.001
Three‐year survival rate (%)	13 (15.1)	42(29.8)	<0.001

### 3.3 Postoperative results

**FIGURE 3:**
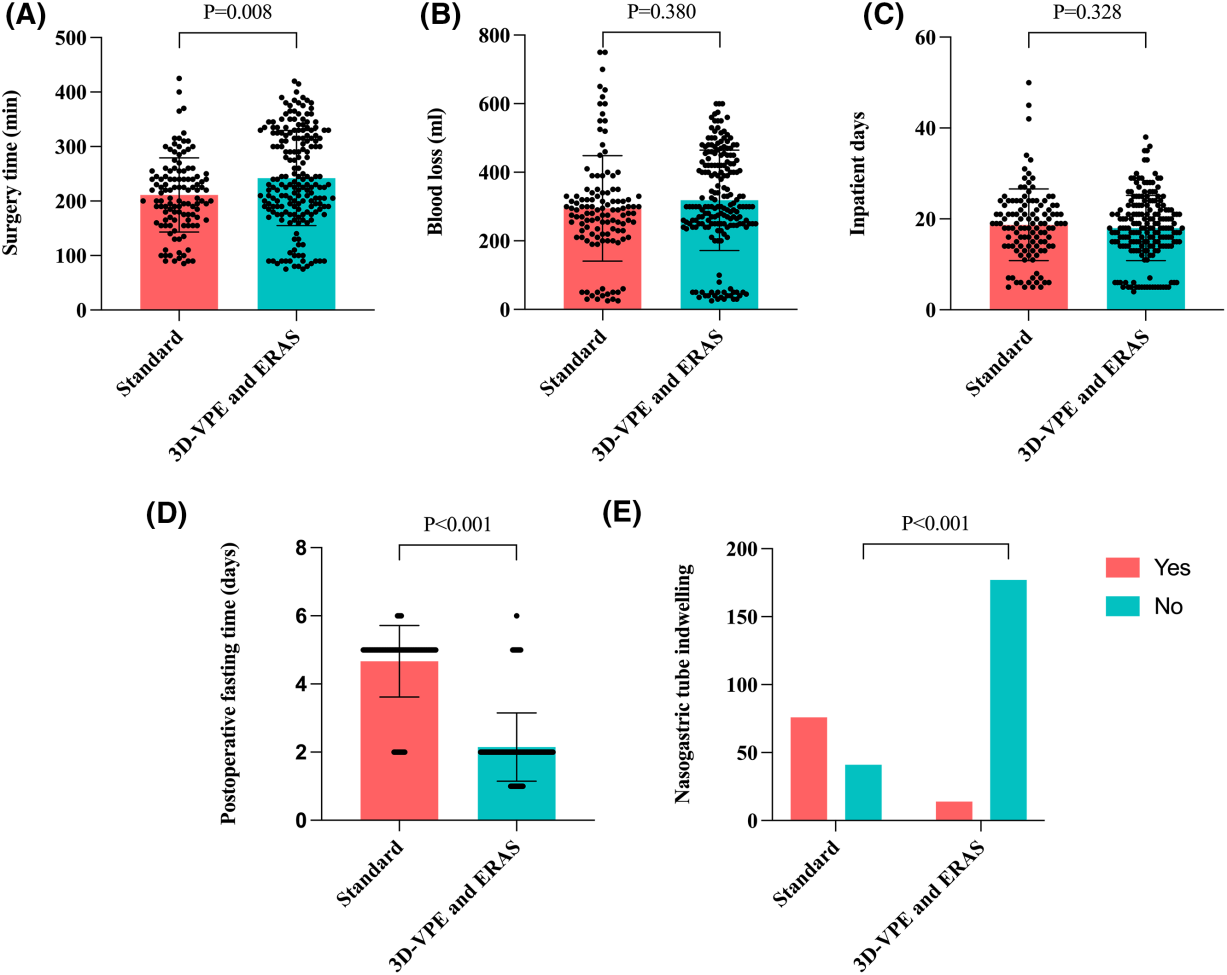
Characteristics of ERAS perioperative management. (A) Comparison of surgery time (min) in two protocols. (B) Comparison of blood loss (ml) during surgery in two protocols. (C) Comparison of inpatient time (days) in two protocols. (D) Comparison of postoperative fasting time (days) in two protocols. (E) Comparison of nasogastric tube indwelling rate (%) in two protocols.

Patients treated with 3D‐VPE and ERAS protocol showed effective pain control, reduced use of several drainage tubes, reduced nasogastric tube indwelling, and fast recovery of early activities (Figure 3), thus demonstrating a reduction in postoperative stress reaction and complications(Table 3).

The median survival time in patients treated with 3D‐VPE and ERAS was 23.1 months (95% confidence interval [CI]: 21.1–25.1), and their 1‐ and 3‐year survival rate were 84.4% and 29.8%, respectively. In contrast, the median survival time in patients treated with standard protocol protocol was 12.7 months, (95% CI: 9.7–15.7) and their 1‐ and 3‐year survival rate were 53.5% and 15.1%, respectively. Evidently, the median survival time and 1‐year survival rates in patients who received the innovative combination management were significantly higher than those in patients treated conservatively with standard protocol (*p* < 0.001, Table 3).

### 3.4 Multivariate analyses of overall survival

**FIGURE 4:**
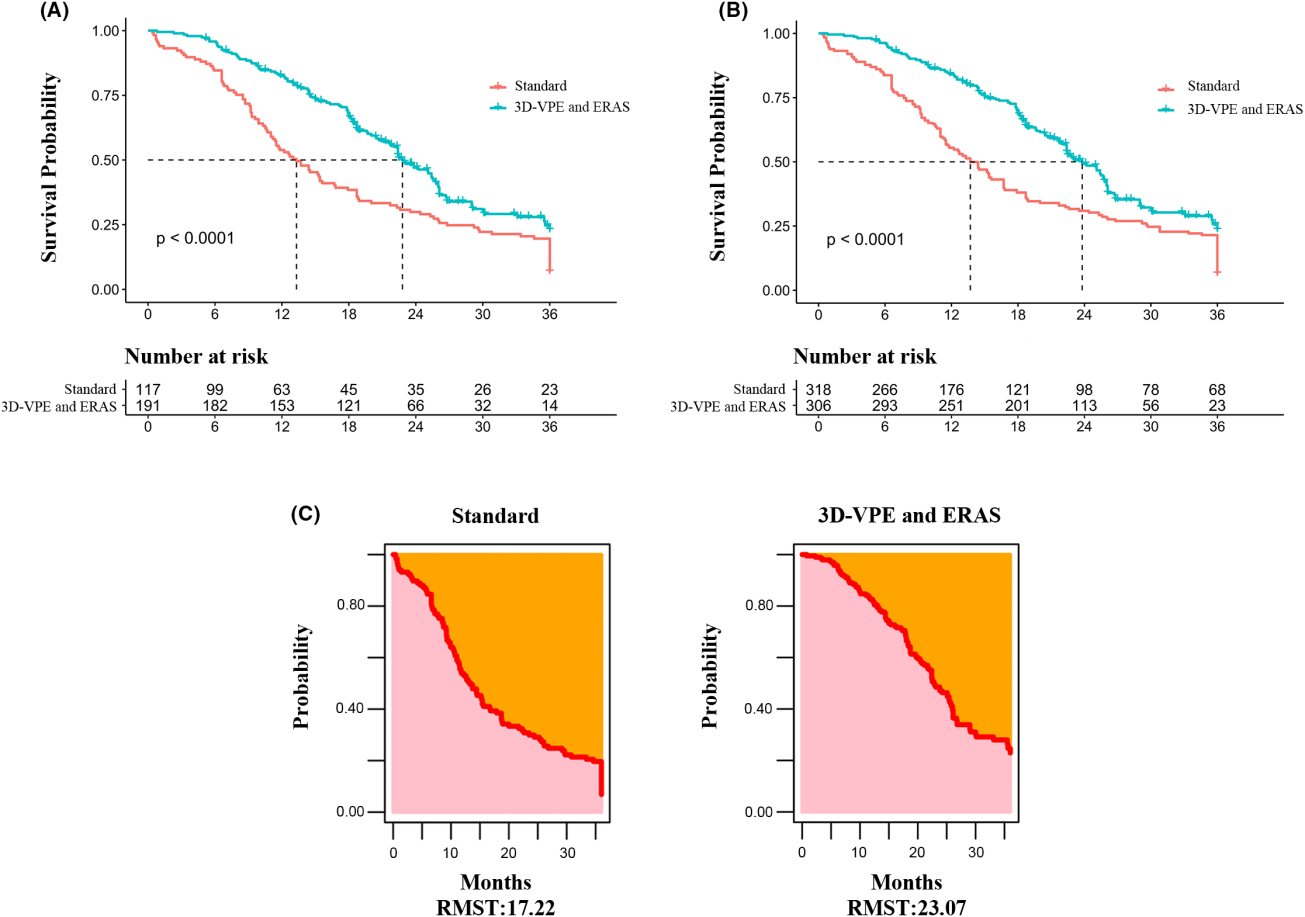
Comparison of the 3‐year overall survival (OS) rates of patients treated in the two protocols. (A) Kaplan–Meier overall survival (OS) estimates. (B) Kaplan–Meier overall survival estimates with inverse probability treatment‐weighting analysis. (C) Restricted mean survival time (RMST) curves for two protocols.

Cox regression analyses showed that age (HR = 1.074, 95% confidence interval (CI): 2.715–5.828, *p* = 0.003), sex (HR = 0.613, 95% CI: 0.443–0.847, *p* < 0.001), TNM stage (HR = 7.110, 95% CI: 4.934–10.245, *p* < 0.001), tumor differentiation (HR = 0.69, 95% CI: 0.515–0.933, *p* = 0.016), and mortality (HR = 3.978, 95% CI: 2.715–5.828, *p* = 0.003) were significant risk factors of overall survival (Table 4). In 3D‐VPE and ERAS group, age, sex, TNM stage, and tumor differentiation were recognized prognostic factors. The survival time of patients treated with 3D‐VPE and ERAS was longer than that of the control group. One may fit OS data with estimate restricted mean survival time (RMST). Significantly improved estimation of survival is 23.07 months for 3D‐VPE and ERAS group and 17.22 months for standard group, with a difference of 5.85 months. (Figure 4). Our results strongly suggest that the introduction of 3D preoperative evaluation, innovation of precise intraoperative pathways, and implementation of perioperative management after 2011 reduced the risk factors associated with GBC surgery and improved patient prognosis.

**TABLE 4:** Results of univariate and multivariate analyses and inverse probability weighting of overall survival.

Characteristics	Total (%)	Mortality (%)	Univariable *p*	Multivariable	
				HR (95% CI)	*p*
Protocol			<0.001	3.978 2.715–5.828	0.003
Standard	86 (37.9)	79 (91.9)			
3D‐VPE and ERAS	141 (62.1)	86 (61.0)			
With inverse probability treatment‐weighting (IPTW)				0.569 0.423–0.765	<0.001
Age (y)			<0.001	1.074 1.055–1.094	<0.001
>60	142 (62.6)	114 (80.3)			
≤60	85 (37.4)	51 (60.0)			
Sex			0.150	0.613 0.443–0.847	0.016
Male	97 (43.2)	70 (72.9)			
Female	130 (56.8)	95 (74.9)			
Gallstones			0.323		
Absent	87 (38.3)	65 (73.7)			
Present	140 (61.7)	100 (74.2)			
Jaundice			<0.001		
Absent	159 (69.8)	102 (66.5)			
Present	68 (30.2)	63 (91.4)			
AFP			<0.001		
>7 μg/L	18 (7.9)	17 (94.4)			
≤7 μg/L	209 (92.1)	148 (70.8)			
CA19‐9			<0.001		
>37 kU/L	130 (57.3)	117 (90.0)			
≤37 kU/L	97 (42.7)	48 (49.5)			
TNM stage			<0.001	7.110 4.934–10.245	<0.001
I	28 (12.3)	5 (17.6)			
II	44 (19.4)	25 (56.8)			
III	109 (48.0)	90 (82.6)			
IV	46 (20.3)	45 (97.8)			
Differentiation			<0.001	0.693 0.515–0.933	0.016
Well	68 (30.0)	34 (50.0)			
Moderately	89 (39.2)	69 (77.5)			
Poorly	70 (30.8)	62 (88.6)			
*Chemotherapy*			<0.001		
Without	105 (46.2)	57 (55.3)			
Gemox	77 (35.7)	72 (93.5)			
AG	27 (11.9)	24 (88.9)			
mFOLFIRNOX	18 (7.0)	12 (66.7)			

## 4 DISCUSSION

Over many decades, traditional surgical management techniques, including 2D imaging technology and personalized surgical pathways, have developed as the standard strategies for GBC resection.^18^ Despite the intense care given to patients with cancer, the high postoperative mortality and reoccurrence rates remain static,^19^, ^20^ the median survival time of patients with GBC remains around 12–16 months, and the 5‐year survival time remains between 5 and 15%. Thus, the innovation of new detection systems and surgical and postoperative management strategies is urgently needed to improve patient outcomes. Here, a novel approach involving the combination of 3D‐VPE and ERAS was evaluated for clinical outcomes and disease prognosis in the cohort treated between 2010 and 2017 and in that treated between 2000 and 2010 (control group). Our results demonstrated that this combined management was associated with an increased R0 resection rate, number of dissected lymph nodes, 1‐year, 3‐year, and overall median survival rates; it was also associated with a decrease in the incidence of some postoperative complications (e.g., biliary fistula) and risk factors (e.g., disease stage and differentiation) relative to the traditional treatment.

Cancer aggressiveness and subsequent metastasis contribute to the primary cause of GBC death.^21^, ^22^, ^23^, ^24^, ^25^, ^26^ First, liver infiltration is common in distant GBC metastasis.^27^, ^28^ To ensure sufficient surgical margins and avoid potential future metastasis, we advocate the expansion of hemihepatectomy or liver resection. Second, lymph node metastasis is also common in aggressive GBC and is an independent prognostic factor in patients.^29^, ^30^, ^31^, ^32^ Regional lymph node dissection is key for successful R0 resection in such cases. By means of 3D visualization technology, we can preoperatively evaluate tumor infiltration into the liver and assess the extent of resection and lymph node dissection required. This technology can locate and distinguish major vessels and bile ducts surrounding tumors, thus helping to avoid damaging the blood supply and ensuring complete blood flow to liver remnants. These suggested approaches can help establish optimal conditions for surgery and prevent disease recurrence.^11^

Radical R0 resection usually involves expanding the resection range and thus increases the potential for postoperative complications; hepatic failure and acute major bleeding of gastric mucosal lesions frequently occur in patients with obstructive jaundice, resulting in postoperative death.^28^ In our proposed management strategy, the optimization of postoperative care can significantly reduce the incidence of postoperative complications. Our supportive regimens include nutrient supplementation, effective pain control, early removal of the nasogastric tube, and decreased postoperative fasting time. All these approaches ensure longer survival times and better quality of life.

In the present study, patients enrolled from the two periods showed differences in diagnoses, presenting symptoms, and non‐perioperative variables that were not statistically significant. The homogeneity of the population in one local area may account for these similarities. Increased albumin, AFP, and CA19‐9 levels were associated with a higher incidence of morbidity; however, these factors were not significant predictors upon multivariate analysis.

However, there were some limitations in this study: (1) Nonrandomized design experiment in a single institution; (2) Full‐ERAS protocol has been gradual completed in the past decade; (3) The sample size was not big enough; (4) Being retrospective in nature. Therefore, given the limits of a nonrandomized study from a single institution, in order to firmly establish this combined management as the standard regimen, increased sample size from multiple cancer centers with randomized trials is essential and thus favorable outcomes of the diseases are expected to benefit GBC patients.

The anatomical specificity and aggressive nature of GBCs pose a challenge for effective surgical treatment; however, the widespread and routine use of the combination of 3D‐VPE and ERAS can pave the way for better surgical and postoperative outcomes for GBC and other cancers as well.

## 5 CONCLUSION

Our study demonstrate the association between implementation of 3D‐VPE/ERAS perioperative management and long‐term outcome of the gallbladder cancer patients with 23.1 and 12.7 months (3D‐VPE/ERAS vs. standard) of median survival time, 23.07 and 17.22 months (3D‐VPE/ERAS vs. standard) of estimate restricted mean survival time. Precision perioperative management implementation including 3D visualization preoperative evaluation and ERAS could benefit patients not only in‐hospital, short‐term outcomes, but also long‐term outcome with gallbladder cancer.

## AUTHOR CONTRIBUTIONS

**Zi‐Yao Jia:** Data curation (equal); formal analysis (equal); investigation (equal); methodology (equal); writing – original draft (equal); writing – review and editing (equal). **Yi‐Di Zhu:** Formal analysis (equal); methodology (equal); resources (equal); writing – original draft (equal); writing – review and editing (equal). **Xiangsong Wu:** Formal analysis (equal); resources (equal); writing – original draft (equal). **Jing‐Xiao Yang:** Data curation (equal); formal analysis (equal); investigation (equal); methodology (equal); writing – original draft (equal). **Wen‐Guang Wu:** Methodology (equal); resources (equal); writing – original draft (equal). **Xu'an Wang:** Methodology (equal); resources (equal); writing – original draft (equal). **Min He:** Methodology (equal); resources (equal); writing – original draft (equal). **Hui Wang:** Methodology (equal); resources (equal); writing – original draft (equal). **Lin‐Hua Yang:** Methodology (equal); resources (equal); writing – original draft (equal). **Jie Zhang:** Methodology (equal); resources (equal); writing – original draft (equal). **Xue‐Chuan Li:** Methodology (equal); resources (equal); writing – original draft (equal). **Lu Zou:** Data curation (equal); investigation (equal); writing – original draft (equal). **Huai‐Feng Li:** Methodology (equal); resources (equal); writing – original draft (equal). **Fei Zhang:** Investigation (equal); methodology (equal); resources (equal); writing – original draft (equal). **Run‐Fa Bao:** Methodology (equal); resources (equal); writing – original draft (equal). **Xu‐Ya Cui:** Data curation (equal); investigation (equal); writing – original draft (equal). **Xiao‐Ling Song:** Data curation (equal); investigation (equal); methodology (equal); writing – original draft (equal). **Wei Chen:** Data curation (equal); methodology (equal); resources (equal); writing – original draft (equal). **Wei Gong:** Conceptualization (equal); project administration (equal); supervision (equal); writing – review and editing (equal). **Maolan Li:** Conceptualization (equal); funding acquisition (equal); project administration (equal); resources (equal); writing – review and editing (equal). **Yingbin Liu:** Conceptualization (equal); funding acquisition (equal); methodology (equal); project administration (equal); supervision (lead); writing – review and editing (equal).

## FUNDING INFORMATION

This study was supported by the National Key Research and Development Program of China (No. 2021YFE0203300), Shanghai Municipal Health Commission health Industry clinical research special project(No.20224Z0014), the National Natural Science Foundation of China (No. 82073206), the Shuguang Program of Shanghai Education Development Foundation and Shanghai Municipal Education Commission (No. 20SG14), the Basic Research Project of Science and Technology Commission of Shanghai Municipality (No. 20JC1419100, 20JC1419101, 20JC1419102), the Clinical Science and Technology Innovation Project of Shanghai Shenkang Hospital Development Center (No. SHDC12019110), Shanghai Clinical Medical Research Center for Digestive Diseases (No.19MC1910200).

## CONFLICT OF INTEREST STATEMENT

The authors declare no conflicts of interest that pertain to this work.

## Data Availability

The data that support the findings of this study are available on request from the corresponding author. The data are not publicly available due to privacy or ethical restrictions.
